# PepScorer::RMSD: An Improved Machine Learning Scoring Function for Protein–Peptide Docking

**DOI:** 10.3390/ijms27020870

**Published:** 2026-01-15

**Authors:** Andrea Giuseppe Cavalli, Giulio Vistoli, Alessandro Pedretti, Laura Fumagalli, Angelica Mazzolari

**Affiliations:** Department of Pharmaceutical Sciences, University of Milan, I-20133 Milan, Italy; andrea.cavalli@unimi.it (A.G.C.);

**Keywords:** protein-peptide docking, machine learning, artificial intelligence, virtual screening, scoring function

## Abstract

Over the past two decades, pharmaceutical peptides have emerged as a powerful alternative to traditional small molecules, offering high potency, specificity, and low toxicity. However, most computational drug discovery tools remain optimized for small molecules and need to be entirely adapted to peptide-based compounds. Molecular docking algorithms, commonly employed to rank drug candidates in early-stage drug discovery, often fail to accurately predict peptide binding poses due to their high conformational flexibility and scoring functions not being tailored to peptides. To address these limitations, we present PepScorer::RMSD, a novel machine learning-based scoring function specifically designed for pose selection and enhancement of docking power (DP) in virtual screening campaigns targeting peptide libraries. The model predicts the root-mean-squared deviation (RMSD) of a peptide pose relative to its native conformation using a curated dataset of protein–peptide complexes (3–10 amino acids). PepScorer::RMSD outperformed conventional, ML-based, and peptide-specific scoring functions, achieving a Pearson correlation of 0.70, a mean absolute error of 1.77 Å, and top-1 DP values of 92% on the evaluation set and 81% on an external test set. Our PLANTS-based workflow was benchmarked against AlphaFold-Multimer predictions, confirming its robustness for virtual screening. PepScorer::RMSD and the curated dataset are freely available in Zenodo

## 1. Introduction

Over the last few years, the interest in peptides as therapeutic entities has increased substantially. The use of these molecular entities dates back to the early 1920s, when the discovery of insulin marked a breakthrough in medicine as an innovative therapy for diabetes [1]. Since then, only in the last two decades, pharmaceutical peptides have found widespread application, with the most prominent areas of interest including metabolic disorders, oncology, and infectious diseases [2,3]. This heightened enthusiasm is reflected in the number of peptide-based approved drugs, which increases yearly [4]. These molecules are characterized by high affinity and specificity for their target, leading to reduced side effects and low toxicity [5]. Their effectiveness derives from the fact that protein–peptide interactions play a crucial role in many biological processes [6], and protein–protein interactions (PPIs) are often mediated by disordered peptide portions [7]. Therefore, an exogenous peptide could mimic such portions, leading to a disruption of the PPI. The main pain points of therapeutic peptides are short half-life, rapid clearance, immunogenic response, limited oral bioavailability, and high production costs [8]. Nonetheless, peptides proved more efficacious than small-molecule compounds when binding to the protein surface or shallow grooves. This is often the case of disrupting PPIs, which can be achieved more easily with peptide binders since the protein–protein interaction surface is often larger than the area covered by small-molecule drugs [9].

Molecular docking is essential in early-stage drug discovery, in particular, in the structure-based drug design of peptide candidate drugs. Several studies have deployed this technique in the last few years to speed up and reduce the costs of the peptide drug discovery process [10,11,12,13,14,15,16,17,18]. Nonetheless, common docking tools, typically trained on small molecules, often fail at docking even short peptides [19]. This can be attributed to two reasons: (1) peptides inherently possess high flexibility, and these programs are not able to explore all their degrees of freedom, therefore not sampling near-native conformations, and (2) the employed scoring functions have been developed for small-molecule compounds, making them poorly suited for handling protein–peptide binding interactions [20]. For these reasons, new peptide-specific docking engines have been developed, including AutoDock Crank Pep (ADCP) v. 1.0 [21], FlexPepDock [22], Haddock [23], CABS-dock [24], and others [25,26,27]. A recent review [28] compared fourteen molecular docking tools, either developed for small molecules or peptide-specific, evaluating their ability to redock peptides ranging from 5 to 20 residues in length. ADCP proved to be the best-performing software, although the results were still worse than those obtained on small-molecule compounds.

Over the past decade, numerous studies have reported the development of machine learning (ML) models to tackle the scoring problem [29,30], a crucial challenge in structure-based drug design. Despite these efforts, the problem remains partially unresolved, particularly in the context of protein–peptide docking. In the standard approaches to developing ML-based scoring functions, the generated model predicts the binding affinity between a peptide ligand and a protein molecule and is trained on experimental affinity data of resolved complexes retrieved from online databases such as PDBbind [31]. In other approaches, the model predicts the root-mean-squared deviation (RMSD) between a given ligand binding pose and the corresponding native one, training the ML algorithm with low- and high-RMSD binding poses. In this way, the model learns patterns within the data that differentiate between a good and a bad pose, which is then useful for pose selection and classification. Various studies have applied this strategy to small-molecule compounds [32], but to our knowledge, only two studies were focused on peptides [20,33]. In the first work, Johansson-Åkhe et al. developed a graph neural network (GNN) able to classify coarse protein–peptide binding poses generated with fast-Fourier-transform (FFT) docking. Therefore, the selected conformations need to be further processed and refined. In the second study, Sanner et al. developed a series of random forest classifiers only for short 2–7-residue peptides, training the models on energetic terms as derived from docking simulations and molecular descriptors.

In light of the need for novel approaches specifically suited for peptide molecules, in this work, we developed PepScorer::RMSD, an ML-based scoring function for the pose-selection task. PepScorer::RMSD was trained and tested using a diverse, high-quality dataset of protein–peptide complexes that we collected and curated from the PDB database [34]. The refined molecular structures were deployed to generate different binding conformations that were then used to calculate multiple sets of features, ultimately helping develop the machine learning algorithms. The performance of the final model, obtained on our evaluation set, was compared in terms of docking power (DP) with other common, machine learning, and peptide-specific scoring functions, reporting remarkable results, with a DP of 96%. Afterwards, we assessed the DP of PepScorer::RMSD on an external test set collected by Sanner et al., obtaining a DP of 23% on the original untreated data and 81% on the same test set after our protocol of data curation, emphasizing the critical dependence of machine learning models on the quality and source of the training data. Finally, we evaluated our workflow—PLANTS-based pose generation followed by PepScorer::RMSD rescoring—against AlphaFold-Multimer 35 predictions. While AlphaFold-Multimer demonstrated superior performance across the full evaluation set, our approach proved to be comparable, and in some cases even more effective, when focusing on complexes with short peptide ligands (fewer than seven residues) or those with low AlphaFold prediction confidence (average peptide pLDDT below 70). Given these results, along with AlphaFold’s substantial computational and storage demands, we believe that PepScorer::RMSD-based reranking of docking-generated poses emerges as a promising and efficient alternative for reranking protein–peptide binding conformations after docking procedures.

## 2. Results and Discussion

### 2.1. Data Analysis

The dataset for this study includes 298 protein–peptide complexes, selected by a rigorous procedure of data curation. It involves single-chain or multiple-chain proteins and glycoproteins retrieved from the PDB database, in complex with 3- to 10-residue peptide ligands. The 3D structures of the complexes were prepared using an optimization and energy minimization protocol (see Section 3 for details). To decrease data redundancy, the optimized complexes underwent a filtering procedure based on protein sequence alignment, resulting in 298 reference complexes. Notably, different amino acid sequences can still adopt similar 3D conformations; therefore, we also conducted a structural similarity analysis focused on the protein binding pockets. This analysis was carried out by the MM-align algorithm [35], which allows the alignment of protein structures composed of multiple segments, and the results are reported in the heatmap in terms of the TM-score (see Figure S1). The plot highlights that the previously performed filtering procedure reduced structural redundancy within the dataset, as only a few similar binding pockets remain. To ensure structural diversity between the training and validation sets and avoid biases in evaluating model performance, we carried out structural clustering of the dataset using a TM-score of 0.4 as the threshold. This means that the complexes in each cluster do not share more than 40% of structural identity. We obtained 126 clusters, many of which included only one entry, as expected, since the previous step of similarity sifting already retained dissimilar sequences. With this procedure, we ensure that no structurally similar peptide–protein complexes can appear in both training and evaluation sets.

### 2.2. Feature Exploration

For each complex, different peptide binding conformations were generated through energy minimization and molecular docking. Each conformation was described by four different types of features: two related only to the peptide and two encoding information about the peptide–protein complex. The peptide was described by a set of known 3D ligand-based molecular descriptors, plus a new kind of descriptor internally developed to evaluate the peptide conformation based on the Φ and Ψ angles of its residues, named the Ramachandran index. The underlying idea of the Ramachandran index descriptors is that, in near-native poses, these angles are likely to adopt certain preferred values, implying a correlation between these features and the RMSD. To validate this hypothesis, we measured the correlation in terms of the Pearson coefficient R for the eight terms of the Ramachandran index descriptor and the RMSD values, and the results are depicted by a heatmap in Figure S2. Both Region 1 and psi prob. showed an appreciable correlation with the target variable (RMSD) of 0.44 and 0.48, respectively, confirming our hypothesis. The peptide–protein complex was described by some energetic interaction scores typically calculated during docking simulations, and some known protein–ligand interaction descriptors named PLEC FPs [36].

### 2.3. Model Building and Optimization

The development of the scoring function describing the peptide–protein interaction is addressed by the generation of a regression model that predicts the RMSD of a given peptide pose with respect to the native one. During model selection, the model performance was evaluated over a 10-fold grouped CV in terms of mean absolute error (MAE) and Pearson correlation coefficient R; particularly, the MAE was chosen as the reference metric for model comparison. Table 1 shows the performances of the generated models (in each box, the MAE is reported above and the Pearson R below).

The first models were developed using only one descriptor family, and the Rescore+ features obtained the best average MAE of 1.99 Å and a Pearson R of 0.61 when submitted to the GB algorithm. The here-reported PLEC SEL. feature set results from a procedure of feature selection obtained by cleaning uninformative features with a variance threshold of 0.1 and then applying the tree-based estimator feature selection with the RF algorithm. The choice of the variance thresholds derives from a study involving three values, as reported in Table S1, which revealed that the cleaning of the PLEC descriptors with growing variance thresholds did not produce relevant changes in model performance, despite the consistent reduction in the feature number.

Later, we combined different descriptors to improve the results, as follows. First, we tested the so-called “Ligand” feature set, which included 3D descriptors selected by correlation analysis (3D CORR) and the Ramachandran index descriptors (Ram.). This feature set outperformed the two features alone, achieving an average Pearson R of 0.55 and a mean absolute error (MAE) of 2.07 Å. Then, the Ligand feature set, the Rescore+ features, and the PLEC SEL. The features were tested two by two in all possible combinations, and finally, all three together. Moreover, as explained in the Section 3, the above-described combined feature sets include the peptide length features obtained through one-hot encoding.

Overall, the feature sets that offered the best performances in terms of average MAE and Pearson R were Ligand plus Rescore+ (line 6, Table 1) and Ligand plus Rescore+ plus PLEC sel. (line 9, Table 1), particularly when used to train RF, GB, and HGB algorithms. To select the best model, we submitted these two input matrices to a protocol of model optimization, including sequential feature selection (SFS) and hyperparameter tuning. The performances obtained during a 10-fold cross-validation after these optimization procedures are displayed in Table 2 in terms of average MAE (above) and R (below). While the SFS generally improved model performance, the tuning did not contribute to this enhancement, and therefore, it was considered avoidable in favor of the scikit-learn v. 1.4.0 default parameter settings. This study also revealed the effective contribution offered by the PLEC descriptors, highlighting their low impact on the model’s predictive ability (an improvement of 0.03 for Pearson R and 0.05 for average MAE when trained with HGB), which did not justify their computational cost. Regarding the algorithm choice, all three methods obtained comparable results, but HGB was much faster in training, and the SFS, in this case, selected the lowest amount of features (30 vs. 38 for GB and 39 for RF). Therefore, HGB trained with Rescore+ and Ligand features selected by SFS was defined as the final best model.

#### Feature Importance Analysis

In sequential feature selection, features selected earlier typically contribute more to model performance. Since the HGB model does not provide feature importances, we analyzed the top-five selected features, sorted as obtained by the selection procedure, to identify those most relevant to model performance. Table S2 reports the 30 sorted features along with their respective Pearson correlation coefficients with the RMSD values. The top-five selected features were the “psi_prob.” from the Ramachandran index descriptors, followed by four Rescore+ descriptors: PLANTS_CHEMPLP_NORM_CONTACT, which is the CHEMPLP score [37] normalized by the number of contacts between the receptor and the ligand; ElectDD, which represents the electrostatic interaction energy calculated using the Coulomb equation and the distance-dependent dielectric constant; the CHARMM score, which expresses the intermolecular interaction energy between ligands and proteins calculated with the CHARMM force field; APBS_Ligand, which represents the electrostatic potential energy of the ligand as calculated using the Poisson–Boltzmann equation.

The analysis of the top-five selected features revealed the importance of the peptide conformation, since the first-ranked one is a structural ligand descriptor, and the fifth Rescore+ descriptor is related to the ligand alone (APBS_Ligand). On the other hand, the estimation of the protein–ligand interaction energy is also relevant to evaluate the goodness of a binding conformation, since ElectDD, CHARMM, and normalized CHEMPLP predict such energy values by using different approaches.

### 2.4. Model Evaluation Results

#### 2.4.1. Internal Validation Performance

The predictive performance of the best model was assessed on an evaluation set including 25% of the starting data. For the effect of the structural stratified clustering, the evaluation set has the same pose composition as the training set: at least two good poses are present for each protein–peptide complex, and the binding pockets of the proteins do not share more than 40% structural similarity with any of the training proteins (TM-score of 0.4). As mentioned in the methods, since some features used to train the model derive solely from peptides, we ensured the absence of biases in the selected evaluation set, verifying that the internal validation performances were not influenced by the peptide sequence identity (Section S1 and Figure S3 of the Supporting Information). PepScorer::RMSD reached, on the evaluation set, an MAE of 1.77 Å and an R of 0.70, in line with the results obtained during cross-validation. This confirms the model’s ability to generalize to unseen protein–peptide complexes.

**Figure 1:**
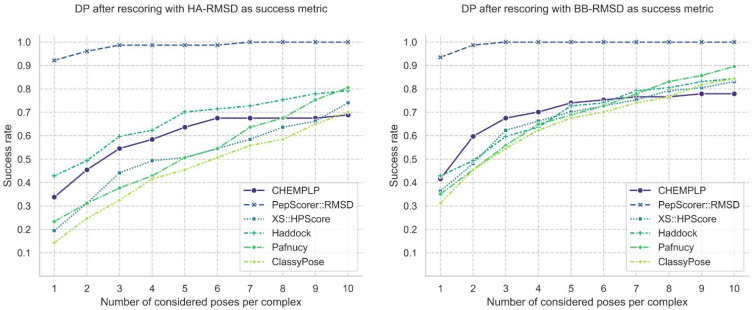
Docking power (DP) of scoring functions across the evaluation set. Success rate is defined as the proportion of successful poses among the top-n ranked (n = 1–10). PepScorer::RMSD achieves the highest top-1 DP (92%), outperforming all tested scoring functions. Small-molecule functions (CHEMPLP, XScore) and ML-based methods (Pafnucy, ClassyPose) show lower performance. The Haddock score, specific to protein–peptide complexes, performs comparably, especially when evaluated using backbone RMSD (BB-RMSD).

To better assess the performance of a scoring function, another evaluation metric known as docking power (DP) was calculated, which measures the ability to detect the native binding mode among decoy poses as the top-ranked solution. The DP of PepScorer::RMSD and of other common and ML-based scoring functions were evaluated for performance comparison. PepScorer::RMSD outperforms all the other tested scoring functions, reporting a DP of 0.92 for both the HA-RMSD and BB-RMSD. This means that the top-1 ranked pose has an RMSD under the threshold for 92% of the complexes (Figure 1). CHEMPLP and XScore [38], representative scoring functions originally developed for docking of small molecules, demonstrated relatively inferior performance in comparison to our method. Notably, ML-based SFs, such as Pafnucy [39] and ClassyPose [40], performed similarly or worse than common ones. For example, ClassyPose is a scoring function developed for the pose selection task that predicts the probability of a given pose being “near-native”. The method exhibited strong performance on the protein–ligand complex dataset curated by the original authors, which excludes peptide-containing structures, achieving a DP of 0.9. In contrast, its performance on our peptide-focused evaluation set was markedly lower, with DP values of 0.14 and 0.31 for HA-RMSD and BB-RMSD metrics, respectively. The HADDOCK score [23], tailored for protein–peptide interactions, exhibited docking performance (DP) comparable to other general-purpose scoring functions such as CHEMPLP. Additionally, we attempted to assess the scoring function developed by Sanner et al. [20] specifically for peptides on our evaluation set; however, technical issues related to file corruption in the publicly available implementation (GitHub: https://github.com/sannerlab/ProtPepRFScorePaper2021, accessed on 17 May 2024) precluded successful execution.

Collectively, the poor performances of existing methods underscore the need for a peptide-specific scoring function optimized for the pose selection task. The strong results achieved by PepScorer::RMSD validate the effectiveness of our peptide docking workflow, which integrates a conventional molecular docking algorithm, namely PLANTS, with a machine learning-based scoring function specifically tailored for the pose selection task.

#### 2.4.2. External Validation Performance

To further validate our model and test it on differently processed data, we used the external test set provided by M. Sanner and collaborators [20], composed of 47 protein–peptide complexes. This dataset presents the following differences compared to our data: (i) It includes peptides with missing residues at both termini of the peptide chain, as well as complexes containing metals and cofactors in the binding site; (ii) it includes peptides with missing oxygen atoms in the C-terminal residue; (iii) during pose generation, the structures did not undergo any energy minimization before the redocking procedure. For this reason, we conducted two different assessments: one involving poses and receptors as supplied by the authors, the other applying the processing protocol designed for our dataset and regenerating the poses by energy minimization and docking simulations. PepScorer::RMSD was benchmarked against the Haddock3 scoring algorithm, selected because it reported the second-best DP on the evaluation set considering HA-RMSD, after our scoring function (Figure 1). The two experiments were later repeated a second time, considering only docking-generated binding conformations.

**Figure 2:**
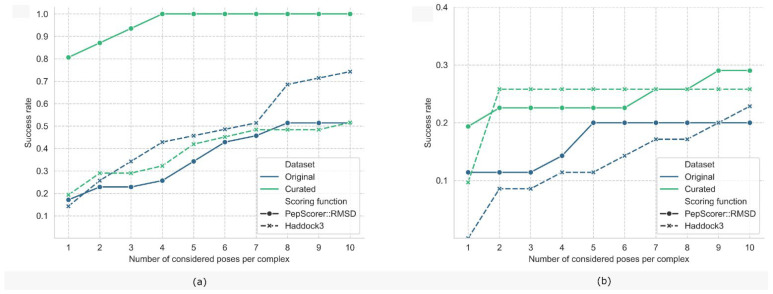
External validation on the pep47 dataset. (**a**) Comparison of two experimental setups. Blue: Docking power (DP) obtained using PepScorer::RMSD and Haddock3 on the original poses provided by M. Sanner and collaborators. Green: DP obtained using PepScorer::RMSD and Haddock3 on curated pep47 poses combined with newly generated conformations. In the first setup, Haddock3 performs comparably to PepScorer::RMSD, whereas in the second, PepScorer::RMSD substantially outperforms Haddock3. (**b**) Results for docking-only binding conformations under the same two setups. PepScorer::RMSD consistently achieves higher top-1 DP than Haddock3 in both cases.

Figure 2a shows the results of both experiments when all poses are considered. On the original dataset (blue lines), our model achieved a top-1 DP comparable to Haddock3 but exhibited a substantial performance drop relative to the DP reported on the evaluation set. As previously noted, the pep47 dataset was curated using less stringent criteria, and its poses include native and minimized conformations, which our model often failed to classify as low-RMSD. This discrepancy likely stems from differences in structure preparation and selection, indicating a dependency of our model on our data curation protocol. In the second experiment (Figure 2a, green lines), PepScorer::RMSD significantly outperformed Haddock3, reaching a top-1 DP of 81% and achieving 100% for the top-4 poses and beyond. It is important to note that both datasets contain X-ray conformations; the only distinction lies in the protein structures, as the curated dataset underwent side-chain energy minimization. The inability of PepScorer::RMSD to identify X-ray poses as low RMSD in the original dataset may therefore be attributed to the absence of side-chain minimization.

Figure 2b reports the DP values of the evaluated scoring functions (SFs) in both experiments, considering only docking-generated poses—a scenario more representative of real-world applications, where X-ray and minimized conformations are typically unavailable. PepScorer::RMSD achieved a higher top-1 DP than Haddock3 across both the original and curated datasets, with an improvement of approximately 10% in each case. Although Haddock3 approaches the performance of PepScorer::RMSD when multiple poses are considered, this finding is particularly relevant because, in virtual screening campaigns, it is common practice to select only the top-ranked solution for further evaluation. Therefore, the top-1 DP represents the most critical metric for assessing a scoring function’s effectiveness in pose selection.

#### 2.4.3. Performance Comparison with AlphaFold-Multimer

AlphaFold 2 (AF2) [41] is widely recognized as the state-of-the-art tool for protein structure prediction, with its variant AlphaFold-Multimer [42] demonstrating strong performance in modeling protein–peptide interactions, while offering a fresh perspective on the challenges of protein–peptide docking. In this study, we investigated the benefits of integrating classical molecular docking with machine learning-based rescoring strategies, as an alternative to fully AI-driven approaches for generating protein–peptide complexes.

An initial observation from the comparative assessments is that AF2, as a purely AI-based approach trained on an extensive dataset of protein–peptide structures, demands significantly greater computational resources and time than our workflow. This limitation configures AF2 as suitable for focused studies aimed at optimizing a limited number of peptide candidates, whereas our method is well-suited for large-scale docking campaigns, including virtual screening applications. Moreover, to realistically interpret the reasons behind AF2’s performance, it is important to note that, although the complexes in the evaluation set were excluded from AF2’s structural templates, the model may have been trained on proteins that are highly similar, or even identical, to those in the evaluation set.

Interestingly, in all three assessments (Figures S4 and S5), AF2 performance remains constant regardless of the number of poses considered. This behavior can be attributed to two factors: (i) AF2 tends to produce structurally similar binding conformation across its predictions, and (ii) under default settings, only the top-ranked AF2 solution undergoes energy minimization, and many unminimized poses are excluded because they fail to meet the chemical and physical validity criteria.

When applied to the full evaluation set, AF2 outperformed the PLANTS plus PepScorer::RMSD workflow, achieving a top-1 DP of 28% (Figure S4a). As expected, our method performed substantially worse than in internal validation, due to the exclusive reliance on docking-generated poses. Performance increased progressively from 7% with a single pose to 18% with ten poses. The second assessment aimed to compare the methods under conditions favorable to our workflow. Given that the PLANTS algorithm struggles to produce a sufficient number of high-quality solutions for longer peptide sequences, we limited the evaluation set to complexes containing peptides of six residues or fewer. Under these conditions, AF2’s top-1 DP dropped to 21%, while our approach achieved a top-1 DP of 18% and reached 43% within the top-10 poses (Figure S4b). These results suggest that AF2 is less effective for shorter peptides, likely due to their tendency to bind to target proteins in an unstructured manner. On the contrary, longer peptides often adopt secondary structure elements that AF2 can identify through multiple sequence alignment and model with greater accuracy. Our scoring function, on the other hand, demonstrates greater effectiveness in pose selection when provided with a sufficiently diverse and valid set of docking solutions. The final assessment explored whether our workflow could serve as a viable alternative for complexes poorly predicted by AF2. In this subset, both methods achieved a top-1 DP of 9%, but PLANTS plus PepScorer::RMSD improved to a top-10 DP of 24% (Figure S5). The limited performance of AF2 correlates with a strong negative relationship between the peptides’ average pLDDT and HA-RMSD (Pearson R = −0.7), whereas the solid performance of PepScorer::RMSD reinforces the relevance and advantages of our integrated workflow.

#### 2.4.4. Applicability Domain Results

**Figure 3:**
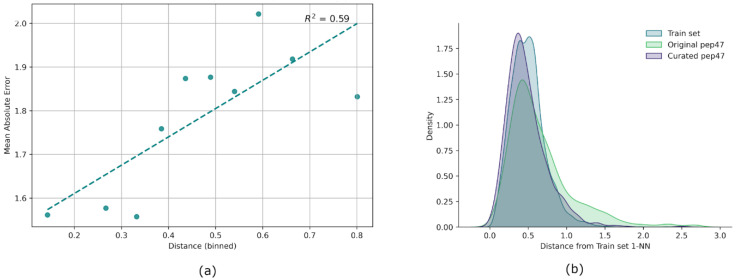
Pose distance analysis. (**a**) Correlogram showing the relationship between binned 1-NN distances and bins’ mean absolute error (MAE), based on training set poses within the 95th percentile. Bins are defined at every 10th percentile, and represented by the dots, while the dashed line illustrates the average trend. (**b**) Probability density curves of pose distances for training, original pep47, and curated pep47 datasets estimated via kernel density estimation (KDE). Training set distances were computed during 5-fold cross-validation.

To assess the reliability of PepScorer::RMSD predictions across its applicability domain, we analyzed the relationship between prediction error and the distance of test poses from the training data, as determined by the 1-nearest neighbor (1-NN) approach. The validation poses were binned based on their 1-NN distances. For each bin, we calculated the mean absolute error (MAE) of the predicted RMSD values during a 5-fold grouped CV. To ensure that the analysis reflected the chemical space explored by the core of our data, we excluded training set outliers, defined as poses with 1-NN distances greater than the 95th percentile (p95) of the training set distance distribution. This effectively resulted in the exclusion of 255 poses out of the 5083 training poses. As shown in Figure 3a, we observed a clear positive correlation between the 1-NN distance and MAE: validation poses that were closer to the training data exhibited lower prediction errors, while more distant poses generally resulted in higher errors. Based on this observation, one can assume that predictions for highly distant poses are less reliable.

Afterwards, we calculated the 1-NN distance between the entire original and curated pep47 datasets and the training set. Figure 3b shows the probability density curves for the training set (represented by the validation set distances calculated during the 5-fold CV) and the two external test sets. The curated pep47 set exhibits a distribution more similar to the training set compared to the original pep47 set. To quantify the similarity between the distributions, a two-sample Kolmogorov–Smirnov (KS) test was performed using SciPy’s ks_2samp function. The KS statistic measures 0.188 for the original pep47 set and 0.094 for the curated version (both with *p* < 0.05). These results indicate a moderate divergence between the training data and the original test set, which may partially account for the lower predictive performance of PepScorer::RMSD on that dataset. Conversely, the curated test set closely resembles the training distribution, which aligns with the improved predictive performance observed on this set.

## 3. Materials and Methods

**Figure 4:**
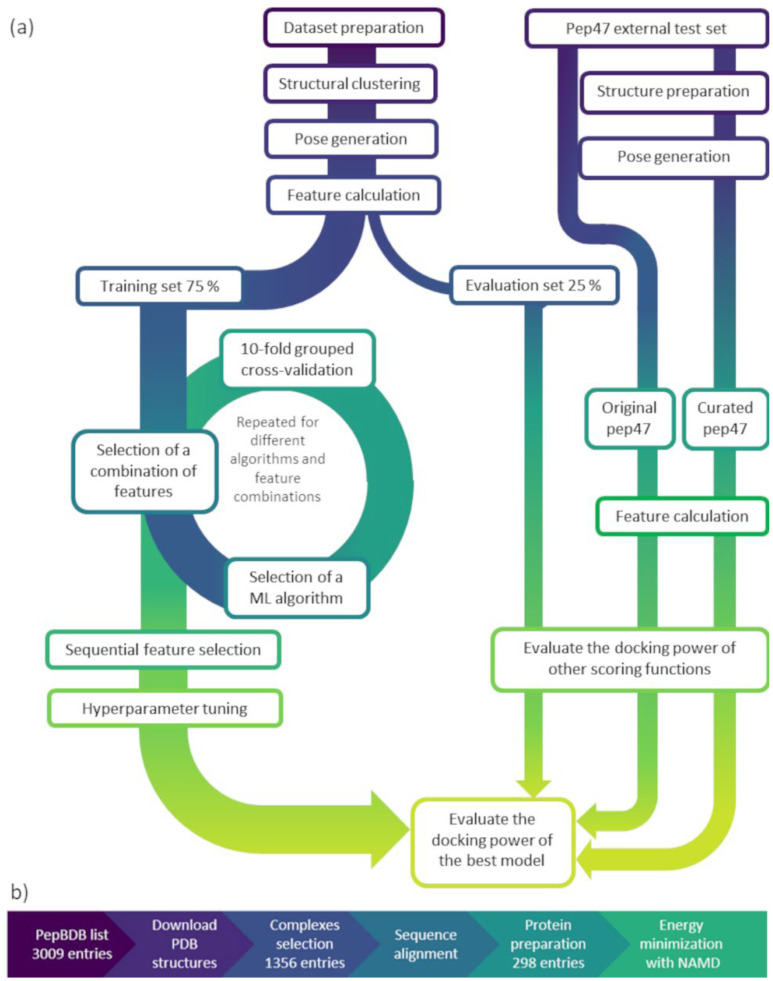
Study workflow and dataset preparation. (**a**) Overview of the study workflow. **Left** panel: Development of PepScorer::RMSD. The dataset was built by collecting and curating protein–peptide complexes, which were then clustered based on binding pocket structural similarity. For each structure, multiple binding poses were generated and described through computed features. Model training involved 75% of the data and included grouped 10-fold cross-validation for descriptor and algorithm selection, sequential feature selection for dimensionality reduction, and hyperparameter tuning. **Right** panel: Evaluation of PepScorer::RMSD through internal validation (25% of the original data) and external validation using the *pep47* test set, both in its original and curated versions. Performance was assessed via docking power (DP) and compared to standard scoring functions. (**b**) Dataset preparation steps. Starting from 3009 entries, 298 protein–peptide complexes were retained for model generation.

The outline of this study includes a protocol for preparing the input matrix, involving pose generation and feature calculation, and a phase of model training and validation for selecting and using the best model, as depicted by the workflow in Figure 4a. The dataset preparation concerned 3009 protein–peptide complexes, with peptides containing 3 to 10 residues, gathered from the RCSB PDB (https://www.rcsb.org), which were progressively filtered to collect a high-quality dataset of 298 final entries (Figure 4b). These complexes, referred to as “reference” complexes, underwent optimization by energy minimization and further curation. The next step concerned the generation of different peptide binding poses for each complex, exploiting energy minimization and molecular docking. For every obtained pose, the root-mean-squared deviation (RMSD) with respect to the relative reference pose was calculated, as well as other ligand- and structure-based features, with the aim of catching different information about the peptide–protein complexes. Afterward, the dataset of peptide–protein poses was divided into training and evaluation sets by a random split into 75–25%, respectively, to obtain a first performance evaluation. Notably, the split was performed after clustering the entries to preserve a certain structural diversity between the two resulting subsets. Different machine learning (ML) algorithms, with varying combinations of features, were internally evaluated on the training set during a nested 10-fold cross-validation to select the final best model based on model performance (mean absolute error (MAE) and Pearson correlation coefficient (R)) and complexity. Later, the best model was tested on the evaluation set, and the model performance was evaluated in terms of MAE, R, and docking power (DP) and compared to other classical and ML scoring functions. Additionally, to further investigate the robustness of our best model, the scoring function’s performance was evaluated on an external test set provided by another research group, which was collected using a different protocol. Two experiments were carried out: one using the original poses provided by the authors, and another with processed structures and newly generated poses.

### 3.1. Input Matrix Preparation

#### 3.1.1. Data Sources and Processing

In this work, we compiled a novel, high-quality, curated dataset of protein–peptide complexes. We focused on short peptides, with a length between 3 and 10 residues. Figure 4b reports the steps of the dataset preparation with the relative size evolution for the number of entries. The protein–peptide complexes were retrieved from the PDB database, according to a list of relevant entries obtained from the PepBDB database [43] (updated in October 2023). Moreover, to include glycoproteins, we also queried the RCSB website (accessed in October 2023) for complexes with a protein/oligosaccharide polymer composition. The search was focused on X-ray crystal structures with a resolution not lower than 3 Å, not presenting non-natural amino acids. A total of 3009 complexes were downloaded and then reduced to 2963 entries after removing duplicates, observed when the same PDB entry was reported multiple times, and structures containing nucleic acids. To identify and select the protein chain/s that interact with the peptide ligand, we submitted the collected PDB files to a Python script that implements the same selection criteria used in the PepBDB database, in which the interaction requires at least one heavy atom of the peptide to be closer than 5.0 Å to another heavy atom of the protein receptor.

Data curation was performed to improve the quality of the collected dataset, as follows: (i) the entries were examined for small molecules near the peptide: complexes were discarded when at least one atom of the peptide was less than 7 Å away (856 entries); (ii) the same filter was applied for metals near the peptide (433 entries); (iii) entries with missing residues in the peptide structure were discarded (1092 entires); (iv) entries with missing atoms in the peptide structure were discarded (392 entires); (v) entries with proteins with less than 500 atoms were removed (105 entries). This procedure reduced the dataset to 1356 entries, less than half of the initial complexes. A substantial portion of the excluded entries corresponded to peptides with missing residues.

We chose to remove these cases for two main reasons: (i) the absence of several atoms in the peptide ligands would make reconstruction highly uncertain, introducing inaccuracies; and (ii) retaining incomplete peptides without modeling the missing residues would yield unrealistic binding poses, as a significant part of the ligand would be absent. The decision to remove complexes containing small molecules or metal ions in proximity to the peptide was motivated by the need to address a simple and well-controlled scenario, where the docking problem involves only two entities: the protein and the peptide. After establishing and validating the simple case, the more complex situation involving a third interacting entity will be addressed in future work. To decrease data redundancy, we performed a sequence alignment using CD-Hit software v. 4.8.1 [44], and we clustered proteins, setting the sequence identity threshold at 0.75. Two analyses were performed, one for single-chain proteins and another for multiple-chain ones. Finally, we selected the representative complexes for each cluster for further visual inspection to check whether the peptide was covalently bound to the receptor, either through a disulfide bridge or a peptide bond. We ended up with 298 high-quality protein–peptide complexes (similar size as by Sanner et al. [20]), which underwent a procedure of dataset preparation.

The selected structures were prepared by exploiting the UCSF ChimeraX [45] functionality “Dock Prep”, using default settings and avoiding hydrogen addition. Missing residues were inserted with the ChimeraX tool “Model Loops,” and the VegaZZ v. 3.2.3 [46] software was used to add hydrogens and ionize acidic and basic groups according to the physiological pH condition of 7.4. Additionally, the carboxylic groups of the peptide ligands were fixed by adding a second oxygen atom, when missing, by means of VegaZZ. The partial charges were calculated with the Gasteiger–Marsili method [47], as implemented in VegaZZ. To relax the complex structures and resolve close contacts, energy minimization was performed with the software NAMD v. 2.14 [48], through VegaZZ, treating the protein backbone and the peptide as rigid and the protein side chains as flexible. Thus, the prepared complexes were considered the reference structures.

#### 3.1.2. Structural Clustering

The structural similarity between training and validation/test sets affects model performance; in particular, a high similarity tends to overestimate the ability of the model to predict novel, structurally diverse proteins [49]. To preserve structural diversity between training and test data during internal validation, we focused attention on the protein structures. Indeed, the final dataset is composed of protein–peptide complexes in which the same protein structure is in complex with different peptide conformations, which realistically prevents the same peptide structure from being present in both training and evaluation sets. This evidence was further confirmed by an analysis of the model performance’s dependency on peptide similarity, as better described in the supporting information (Section S1 and Figure S3).

Therefore, to accomplish stratified splitting during model validation, we performed structural clustering of the protein binding pockets, as defined by a 10 Å sphere around the peptide ligand. This was achieved with the software MM-align v. 20210816 [35], which allows the alignment of protein structures composed of multiple segments. MM-align first joins the segments of each input structure in a single chain and then performs the alignment. The software returns two TM-scores for the structure identity: two metrics in the range of 0 to 1, normalized on the length of the two aligned protein chains, respectively. We decided to use the metric normalized by the longer chain. Afterward, these MM-align results were analyzed by a Python v. 3.12 script, based on NetworkX v. 2.8.8 [50], which performs a graph-based clustering with a similarity threshold of 0.4. This was achieved by considering each protein binding site as a node of a graph, and proteins with a TM-score > 0.4 were connected through an edge. Through this procedure, we obtained 126 clusters, which were exploited for stratified splitting applied to both the nested 10-fold cross-validation and the following single evaluation set (75–25%).

#### 3.1.3. Pose Generation

The generation of ML models able to evaluate the goodness of the ligand binding conformation requires model training on a wide and well-stratified range of “good” and “bad” poses, meaning conformations with an RMSD with respect to the native one in the range of 0–20 Å. This can be achieved by exploiting the redocking procedure of the crystallographic ligand into its binding pocket [51]. However, due to the poor performance of docking tools working on peptides, other strategies are needed to enrich the dataset of good poses. Bad poses are easier to obtain, but importantly, should not be too obvious, i.e., they should establish interactions with the binding site that can be energetically favored.

To collect the first good poses, we started with the native conformation of each peptide. Then, we generated some poses by performing two different energy minimization procedures with NAMD: the protein backbone as fixed, the protein side chains as free to move, and the peptide either as free or with a constraint of 0.5. In this way, we obtained two low-RMSD poses for each complex, named “*freelig*” and “*05lig*”, with average RMSD and standard deviation equal to 1.100 ± 0.468 Å and 0.529 ± 0.192 Å, respectively. Figure S6 in the supporting information shows the line plot reporting the RMSD values for *freelig* and *05lig* poses of the 298 complexes.

The redocking procedure was leveraged by setting the native conformation as the input structure to foster low-RMSD poses. The chosen docking programs were PLANTS v. 1.2 [37], a common small-molecule docking software, and ADCP v. 1.0 [21], a peptide-specific docking software. The latter was exploited to increase the number of valid results for longer peptides and was employed only for peptides longer than or equal to 7 residues, since PLANTS was not able to generate enough good solutions in these cases. Regarding the PLANTS parameters, the center and radius were calculated with the “--mode bind” command, using the peptide ligand as input, the cluster RMSD was set to 2 Å, and CHEMPLP was the scoring function of choice. On the other hand, for ADCP, the docking box was identified through AGFR v. 1.2 [52], the number of runs was set to 10, and the number of iterations was 1 million. The two docking engines were set to generate 20 and 10 poses, respectively, but for some complexes, the number of conformations collected was poorer than expected, particularly for longer peptides. In these cases, the number of ADCP poses was raised to not less than 4 by performing energy minimization of the ligand alone before the redocking and, for the harder examples, adding a preparative conformational search with AMMP software v. 2.4.0 (1000 iterations, random method) before the minimization. The ADCP output conformations were prepared to be submitted for further analyses as follows: (i) the missing oxygen atom of the C terminal carboxylic group was fixed through a Python script using RDKit module v. 2023.09.2 (https://www.rdkit.org); (ii) the ionizable groups of the complex were simulated as compatible with a pH condition of 7.4 with the Vega ZZ v. 3.2.3 software; (iii) atom types and charges were assigned by CHARMM and Gasteiger algorithms, respectively.

**Figure 5:**
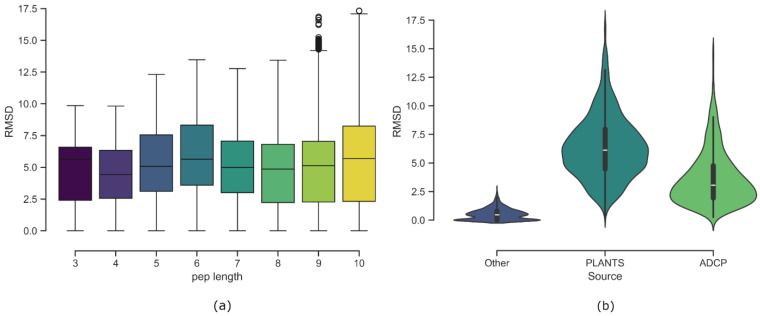
RMSD distribution of collected poses. (**a**) RMSD distribution by peptide length. Each peptide has 23 poses. No clear correlation between peptide length and RMSD is observed: median RMSD values remain around 5 Å, with a peak for 6-residue peptides. Longer peptides show greater RMSD variability. (**b**) RMSD distribution by pose source. “Other” includes native and minimized poses, all with RMSD < 2.58 Å, most < 0.8 Å. PLANTS contributes the widest RMSD range (0–17.3 Å), with most values between 5 and 7.5 Å. ADCP enriches the dataset with low-RMSD poses (2–4 Å).

The heavy-atom RMSD of each pose was calculated with the RDKit function “CalcRMS”, which uses substructure matching to discard the conformations that did not present a match with the native one, except for a few of them where RDKit failed and was replaced by the function “*rmsd*” from the “*spatial*” module of the Python library ODDT [53] v. 0.7. Based on these RMSD values, the best 23 poses were selected for each protein–peptide complex. This number corresponds to the maximum number of poses obtained for the complexes for which ADCP docking was not conducted: one X-ray pose, two minimization poses, and 20 PLANTS poses. The distributions of RMSD values by peptide length, in the form of a boxplot, are reported in Figure 5a. The violin plot in Figure 5b shows the distribution of RMSD values by source, highlighting that the addition of ADCP poses allowed a better coverage of the RMSD range between 2 Å and 4 Å.

#### 3.1.4. Feature Calculation

To best describe each binding pose, four families of descriptors were exploited: (1) 3D ligand-based molecular descriptors, calculated with the Python module Mordred v. 1.2.0 [54]; (2) Ramachandran index descriptors, related to the Φ and Ψ angles of the peptide residues; (3) energetic scores, calculated with the tool Rescore+ [55] in Vega ZZ; (4) protein–ligand extended connectivity (PLEC) fingerprints (FPs) [36] obtained with the Open Drug Discovery Toolkit (ODDT) [53]. Table 3 reports the main calculated features for each descriptor family.

**Figure 6:**
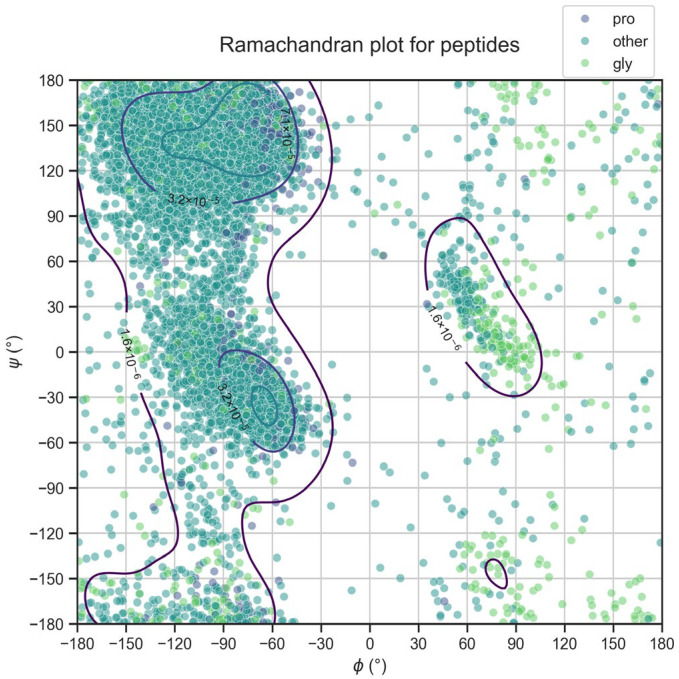
Ramachandran angles of peptides. Scatter plot of Φ (x-axis) and Ψ (y-axis) angles for the 298 peptides. Φ angles are reported on the x-axis; Ψ angles are reported on the y-axis. The distribution resembles a typical Ramachandran plot for protein residues and was used to derive Ramachandran index features.

1. Mordred is a Python package that yields a total of over 1800 molecular descriptors, including 2D and 3D ones. A set of 3D geometrical descriptors was selected and used as such, and normalized by the number of heavy atoms and the number of rotatable bonds, obtaining a total of 147 features. Mordred calculates many versions of the same descriptor, and Table 3 reports only the names of the main different 3D descriptors. The complete list of calculated descriptors is disclosed in Table S3.
2. The Ramachandran index descriptors were developed by us and calculated with a Python script, making use of the Biopython module v. 1.83 [56]. The idea originates from the ADCP scoring function, which includes a component that evaluates the peptide conformation based on the Φ and Ψ angles of its residues [21]. Our descriptors include eight terms for each peptide: four related to 4 regions on the Ramachandran plot of the given peptide, namely “region 1”, “region 2”, “region 3”, and “region 4”, and four related to mathematical elaborations of the Φ and Ψ angles of each peptide residue, namely “Φ mean”, “Ψ mean”, “Φ prob”, and “Ψ prob”. The terms related to the Ramachandran regions are calculated as follows:
  (1) $$R_{i}=\frac{n_{i}}{N}$$
  where $R_{i}$ is the descriptor value referred to the region i (with i being 1, 2, 3, or 4), $n_{i}$ is the number of residues that fall into the specific region i, and N is the total number of residues of the peptide. The four mentioned regions are defined on the Ramachandran plot of peptides (Figure 6) as follows:

Region 1:

i. Φ from −130 to −50 degrees and Ψ from 120 to 180 degrees.
ii. Φ from −75 to −60 degrees and Ψ from −25 to −50 degrees.

Region 2:

i. Φ from −150 to −45 degrees and Ψ from 100 to 180 degrees.
ii. Φ from −90 to −45 degrees and Ψ from 0 to −65 degrees.

Region 3:

i. Φ from −180 to −30 degrees and Ψ from −180 to 180 degrees.
ii. Φ from −30 to 105 degrees and Ψ from −30 to 90 degrees.

Region 4: all the residues not included in any other region.

The Φ and Ψ angles’ values of the regions’ boundaries were chosen as follows: the two-dimensional distribution of these angles was estimated using kernel density estimation (KDE) (reported in Figure 6), which yielded contour lines enclosing regions of high conformational density. For each contour, we defined a bounding box that approximately corresponds to the minimal rectangle in the (Φ, Ψ) space that fully contains the contour. The next features included in the Ramachandran index are Φ mean and Ψ mean, which correspond to the averages of the Φ and Ψ angles of each peptide residue. Finally, the last two features were obtained by computing the averages of the probability densities of the residues of each peptide from the probability density functions (PDF) of the Φ and Ψ angles. These functions were obtained by passing all the Φ and Ψ values of all the peptides initially retrieved from the PepBDB database to the Scipy “gaussian_kde” function, which estimates the PDF via kernel density estimation.

3. The tool Rescore+, as implemented in Vega ZZ, allows the calculation of a wide range of scores on ligand–protein complexes, most of which are associated with common docking scoring functions. In total, 30 scores were selected for this study: the 11 main scores are listed in Table 3, while the complete list is reported in Table S4.
4. PLEC FPs, developed by Wójcikowski and collaborators, demonstrated their ability to accurately describe protein–ligand interactions, as already reported in their original work [36] and in a recent one [40], in which the authors developed a classification model for ligand pose selection. We calculated them by setting default parameters for the fingerprint size (16,384), the ligand depth (2), and the protein depth (4).

To account for peptide length, in addition to these four descriptor families, we included a set of features involving one-hot encoding (from 3 to 10 residues, for a total of 8 features). These features were used only in addition to the combined feature sets (lines 5–9, Table 1).

### 3.2. Model Building

Model building was implemented using Python scikit-learn v. 1.4.0 along with Python libraries pandas v. 2.1.4, numpy v. 1.26.2, matplotlib v. 3.8.2, and seaborn v. 0.13.1. Before any model development, a grouped split was performed to divide the dataset into a training (75% of data) and an evaluation set (25% of data). Data splitting was implemented with the function “StratifiedGroupKFold” of Python scikit-learn, using 4 as the number of splits, the peptide length as the stratification variable, and the structural clusters as groups. In this way, no protein binding site in the evaluation set possesses a structural identity higher than 40% with those in the training set. Additionally, the stratification allowed a similar peptide length distribution to be obtained between the two partitions. An analysis of the RMSD distribution in the two sets for peptide length can be found in the supporting information (Figure S7). With this procedure, we collected 221 proteins to carry out model training and 77 proteins for model evaluation.

#### 3.2.1. Model Selection

The model building involved a model selection phase to select the best-performing algorithm with the best feature combination. These models were evaluated during a nested grouped 10-fold cross-validation in an inner loop working on the training data (the circle in Figure 4a). In this study, we treated the pose selection task as a regression problem; therefore, we employed common regression algorithms to fulfill such an assignment. In detail, the exploited algorithms can be divided into three classes: linear algorithms (linear regression and elastic net regression), tree-based algorithms (random forest (RF), gradient boosting (GB), and histogram gradient boosting (HGB)), and support vector machine (SVM) models (epsilon-support vector regression (SVR)), all trained with default parameters. We constructed models with different feature sets to better understand the contribution and performance of each feature type. Each feature type was used either alone or in combination with other feature types; all the combinations and performance metrics are reported in Table 1. The model evaluation was returned in terms of average mean absolute error (MAE) and Pearson correlation coefficient (R), and between the two, the MAE was the primary metric for the best model selection.

#### 3.2.2. Feature Cleaning

During model selection, two descriptors were preprocessed: Three-dimensional molecular descriptors and PLEC fingerprints. Three-dimensional descriptors were reduced to 46 by removing the collinear features with a Pearson correlation coefficient higher than 0.9 and renamed as “3D SEL.” PLEC FPs were subjected to a multistep procedure as follows. First, low-variance features were cleaned by trying different variance thresholds (0.01, 0.1, and 0.5). Then, those selected with the threshold of 0.1 and 0.5, the threshold of 0.01 was excluded since it reduced the number of fingerprints by a small amount (from 16,384 to 12,294), without causing any increase in performance, were submitted to tree-based feature selection. This method relies on a tree-based estimator, in our case random forest, which assigns feature importances during the training. The features with an importance higher than a threshold are selected, and a model with only those features is constructed. In detail, we used the following thresholds as cutoff values: 0.001, 0.005, 0.01 plus the mean, and the median of all feature importances. The model constructed with PLEC selected with a variance threshold of 0.1 and an RF importance threshold of 0.001 obtained the best average MAE in 10-fold cross-validation. Additionally, this procedure reduced the number of bits in the fingerprints from 16384 to 140. We renamed the PLEC FPs selected in such a way as “PLEC SEL.”. Table S1 shows MAE and R of the models trained with full PLEC and PLEC selected with different variance thresholds, while Table 1 reports the performance of models trained with PLEC SEL. features.

#### 3.2.3. Model Optimization

Based on the average MAE in cross-validation, the best combinations of features and ML algorithms resulting from the model selection phase were submitted for further model optimization, consisting of sequential forward feature selection (SFS) followed by hyperparameter optimization. The SFS was performed with the Python library MLxtend v. 0.2.7 [57], over a 10-fold grouped cross-validation for each subset of features. The parameter “k_features” was set to “best” to select the feature combination reporting the best performance in terms of MAE. With an input matrix optimized by the SFS, the hyperparameter tuning was performed during a 10-fold grouped cross-validation. The optimized hyperparameters and relative search spaces are reported in Table S5. This procedure, together with considerations on model complexity/performance tradeoff (see Section 2 allowed the selection of a single ML model that was further evaluated by internal and external validation.

### 3.3. Model Evaluation

#### 3.3.1. Internal Validation

During internal validation, the performance of the best model selected through the above-described steps was evaluated on the evaluation set previously obtained by splitting 25% of the collected data. This evaluation was measured in terms of MAE and R to confirm the results obtained in cross-validation. Afterward, our model performance on the evaluation set was measured in terms of docking power (DP) to compare it with other scoring functions: PLANTS CHEMPLP, XScore::HPScore [38], Haddock3-score [23] (HADDOCK3, Bonvin’s Lab, https://github.com/haddocking/haddock3, 2022; accessed on 17 May 2024), Pafnucy [39], and ClassyPose [40]. The DP measures how effectively a scoring function identifies a correct pose as the top-ranked solution, calculated as the percentage of complexes in which at least one top-ranking pose has an RMSD below a defined threshold—typically 2.0 Å—relative to the native structure. Here, we computed both the HA-RMSD and the backbone atom RMSD (BB-RMSD) in two distinct analyses. We excluded interface-based metrics, such as interface-RMSD (I-RMSD), DockQ [58], or I-INF [59], because protein interface residues were not modeled, and the peptides are relatively short. The selected scoring functions were used to rescore the peptide–protein-generated poses included in the evaluation set. As a success metric, an HA-RMSD threshold of 2.0 Å and a BB-RMSD of 2.5 Å were adopted, since it is common practice for peptides, and the analysis was carried out by calculating the success rate on a growing number of top-ranked poses, from one to ten.

#### 3.3.2. External Validation

The performance of the best model was also evaluated on an external test set in terms of DP. As an external test set, we used a dataset composed of 47 protein–peptide complexes retrieved from the RCSB PDB by M. Sanner and collaborators [20], referred to as the “pep47” dataset. Many other benchmark sets exist, such as PepPro [60], but most are not focused on short peptides (3–10 residues) and do not provide ready-to-use binding poses for rescoring. In particular, PepPro includes only 23 out of 89 complexes within the acceptable length range, while 47 complexes feature peptides longer than 15 residues. Testing our model on such a dataset would yield unreliable results, as most entries fall outside the model’s domain. On the other hand, the pep47 dataset contains only peptides shorter than 10 residues and provides predicted binding conformations, making it more suitable for our evaluation. We performed two different experiments. The first assessment involved the dataset of poses as provided by the authors on their GitHub repository (https://github.com/sannerlab/ProtPep37_2021, accessed on 17 May 2024). These provided poses (original pep47) were obtained by exploiting redocking by the ADFR software v. 1.0, energy minimization, and neighborhood search. Additionally, the original X-ray structures were also included; therefore, each complex contains at least one successful pose, with an RMSD lower than 2 Å. For this experiment, we cleaned out complexes already present in our dataset and those with peptides composed of only two residues, since the Ramachandran index features could not be calculated, reducing the protein–peptide complexes to 36. The second assessment required regenerating the poses after data processing (curated pep47). In this case, we checked the 36 complexes for the presence of metals or small molecules near the peptide, reducing the data size to 31 entries, and then we submitted them to the processing protocol designed for the training data: we generated 23 poses of each complex through energy minimization and docking with PLANTS, not using ADCP, since the peptide length did not exceed 6 residues. In both experiments and assessments, the performance of PepScorer::RMSD was compared with that obtained by Haddock3. The datasets of poses were rescored with the two scoring functions, and the ranked poses underwent HA-RMSD calculation to compute the DP. The two experiments were repeated after excluding the X-ray and near-native conformations, retaining only docking-generated poses. These conditions better emulate real-world scenarios and provide a more rigorous evaluation of the scoring function DP.

#### 3.3.3. Benchmarking Against AlphaFold-Multimer

Our workflow, which integrates PLANTS docking followed by PepScorer::RMSD rescoring, was further benchmarked against AlphaFold-Multimer [42], an extension of AlphaFold 2 (AF2) [41] capable of predicting protein–protein and protein–peptide interactions [61]. We did not employ AlphaFold 3 (https://alphafoldserver.com/, accessed on 23 September 2025) [62] because this last version is not available as open-source software, and its web server limits users to only 30 job submissions per day, making it impractical for virtual screening. For this assessment, we leveraged the peptide–protein complexes employed for internal evaluation as a benchmark set. Our workflow generated 40 binding poses by PLANTS docking, initiated from two peptide conformations (α-helix and β-sheet, 20 poses each), using the same software parameters as those applied during training pose generation; for AF2, we configured the algorithm to generate 4 predictions per model using 4 distinct random seeds, yielding a total of 20 predicted structures. To prevent AF2 from referencing known templates, we added the 77 evaluation PDB entries to its obsolete PDB list, so that AF2 cannot use them as a structural template. The full BFD database was used [63,64], and the “--max_template_date” flag was set to 2022-05-14; all other parameters adhered to the default settings of AlphaFold v. 2.3.2. In this way, only the top-ranked model was relaxed through energy minimization. Given the difficulties of AI-driven structure-prediction methods in generating chemically and physically plausible poses, to verify the validity of the generated complexes, we employed the PoseBusters’ Python package [65] v. 0.4.6. For both methods, a pose was considered successful if it satisfied two criteria: an HA-RMSD ≤ 2 Å and full compliance with all PoseBusters validation checks. Three distinct assessments were performed: the first utilized the full evaluation set comprising 77 complexes; the second focused on a subset of 28 complexes containing peptides with six or fewer residues; and the third targeted the most challenging cases for AF2, those with a pLDDT score below 70, the threshold commonly used for reliable predictions for AlphaFold’s internal confidence metric.

#### 3.3.4. Applicability Domain Study

The applicability domain (AD) of PepScorer::RMSD was assessed using the 1-nearest neighbor (1-NN) approach [66], where the similarity of a test pose to the training set is measured by its distance to the closest training pose. The 1-NN approach was implemented via Python thanks to scikit-learn’s function “NearestNeighbors”. Since AD analyses are typically performed in ligand-based studies, Euclidean distances between poses were calculated using the eight Ramachandran index descriptors, which depend solely on the peptide ligand conformation. To evaluate whether the employed metric was a reliable indicator of model prediction confidence, we performed a 5-fold grouped cross-validation, computing the correlation between the binned 1-NN distances of validation folds and the mean absolute error (MAE) within each bin.

## 4. Conclusions

We presented PepScorer::RMSD, a novel machine learning-based scoring function trained to rerank protein–peptide binding conformations after docking procedures. The model predicts the RMSD between a given binding pose and the corresponding native one. The main advantage of our method lies in the rigorous protocol for data curation followed when collecting and preparing the training dataset of protein–peptide complexes. By filtering the PDB database, 298 complexes with peptides 3- to 10-residues in length were selected and manually curated. Notably, the dataset is characterized by high sequence diversity and is annotated for binding site structure similarity to gain better generalization performance. For each dataset structure, a set of binding poses was generated through energy minimization and molecular docking. The binding poses were comprehensively described by four types of descriptors, among which the Ramachandran index descriptor was developed by us to evaluate the peptide conformation based on the Φ and Ψ angles of its residues, and proved relevant for model performance during sequential feature selection. Interestingly, the performance of the models generated using only ligand features is not as far as expected from that of models incorporating both ligand and protein information (R = 0.59 compared to R = 0.70), highlighting the importance of peptide conformation in docking engines. These results suggest that integrating the Ramachandran index descriptors developed by us within other existing docking scoring functions may offer a cost-effective yet promising strategy worth exploring in future studies. Among the tested machine learning algorithms, only slight differences have been highlighted in terms of performance, but histogram-based gradient boosting (HGB) was much faster in training. The best-performing model during internal validation was trained on the ligand descriptor set plus the Rescore+ and PLEC descriptors by the HGB Regressor (line 9, Table 1). Following further optimization of the model, we selected the HGB Regressor trained using ligand descriptors and the Rescore+ feature set, excluding PLEC descriptors, as the final configuration. This choice ensures full model interpretability and reduces computational overhead without compromising predictive performance. The finalized model, PepScorer::RMSD, achieved a Pearson correlation coefficient of 0.70 on the evaluation dataset.

We also assessed the docking power (DP) of PepScorer::RMSD and compared it to common, machine learning, and peptide-specific scoring functions. Our model outperformed all the others with a top-1 docking power of 92%. Additionally, to evaluate our model’s robustness, we validated PepScorer::RMSD on the protein–peptide complex dataset collected by Sanner et al. [20]. When exploiting the poses provided by the authors, our model exhibited comparable performance to the other tested scoring function, even if it was lower than during internal validation. Upon reprocessing their complex structures and reproducing the binding poses according to our protocol, predictive accuracy improved significantly, achieving a top-1 DP of 81%. These findings confirmed the importance of data curation in ML modeling and highlighted that (similarly to other ML-based scoring functions) the model produces interesting results when applied for the rescoring of poses derived from the same docking software used to generate the training poses. However, these results do not emulate a real-world scenario, in which only docking-generated poses are scored. Therefore, we repeated the two experiments, considering only docking-generated conformations. In this case, PepScorer::RMSD reported a better top-1 DP than Haddock3, highlighting its potential for post-docking pose selection in virtual screening campaigns. Finally, we compared the DP obtained by reranking PLANTS-generated poses using PepScorer::RMSD with that of AlphaFold-Multimer (AF2). While on the entire evaluation set, our approach fell short, when considering complexes with short peptides (less than 7 residues) or complexes for which AF2 reported low-quality structures (peptide’s average pLDDT < 70), we were able to produce similar results in top-1 DP and significantly better results in top-10 DP (21% vs. 43% for the first case, and 9% vs. 24% in the second).

These findings indicate that PepScorer::RMSD is a reliable tool for ranking protein–peptide binding conformations, with performance largely unaffected by protein structural diversity. Additionally, PLANTS docking followed by PepScorer::RMSD pose reranking can be a valuable substitute to AF2 protein–peptide structure prediction, especially for particularly short peptides and poorly predicted complexes, and the most appropriate solution for virtual screening campaigns. Future work will focus on expanding the training dataset and incorporating a broader range of pose generation strategies to enhance the model’s generalizability across diverse input conditions.

## Figures and Tables

**Table 1:** Combinations of ML algorithms and peptide descriptors used during model selection. Each cell displays the model’s performance, with the average mean absolute error (MAE) shown at the top and the Pearson correlation coefficient (R) at the bottom. Cell colors indicate the MAE values.

			Algorithms				
			Linear Regression	Random Forest	Gradient Boosting	Histogram Gradient Boosting	Support Vector Regression
**Features**	1	Ramachandran index	2.20 0.48	2.25 0.46	2.16 0.50	2.24 0.47	2.10 0.51
	2	Three-dimensional SEL.	2.36 0.40	2.39 0.37	2.38 0.38	2.44 0.37	2.33 0.39
	3	Rescore+	3.51 0.37	2.01 0.59	1.99 0.61	2.01 0.60	2.15 0.52
	4	PLEC SEL.	2.22 0.52	2.37 0.42	2.35 0.44	2.30 0.48	2.29 0.45
	5	Ligand	2.15 0.53	2.10 0.56	2.12 0.54	2.13 0.54	2.07 0.55
	6	Ligand− Rescore+	2.81 0.52	1.81 0.67	1.84 0.66	1.82 0.67	1.95 0.61
	7	Ligand− PLEC SEL.	2.02 0.64	2.04 0.59	2.01 0.61	1.99 0.62	2.12 0.56
	8	PLEC SEL.− Rescore+	3.63 0.47	1.96 0.63	1.92 0.66	1.92 0.66	2.23 0.52
	9	Ligand−PLEC SEL.−Rescore+	2.99 0.56	1.80 0.68	1.79 0.69	1.79 0.70	2.06 0.60

**Table 2:** Model performance after sequential feature selection (SFS) and hyperparameter tuning (HPT). Each cell reports the average mean absolute error (MAE) at the top and the Pearson correlation coefficient (R) at the bottom, based on 10-fold cross-validation. Cell colors represent MAE values.

Features	Method	Random Forest	Gradient Boosting	Histogram Gradient Boosting
Ligand− Rescore+	Base	1.81 0.67	1.84 0.66	1.82 0.67
	SFS	1.75 0.69	1.75 0.70	1.73 0.70
	HPT	1.75 0.69	1.75 0.70	1.74 0.70
Ligand−PLEC SEL.−Rescore+	Base	1.80 0.68	1.79 0.69	1.79 0.69
	SFS	1.72 0.70	1.71 0.71	1.68 0.73
	HPT	1.72 0.70	1.71 0.71	1.70 0.73

**Table 3:** Overview of the main computed features, grouped by descriptor type. A complete list of features is available in the Supporting Information (Tables S3 and S4).

PEPTIDE DESCRIPTOR	CALCULATED FEATURES
RAMACHANDRAN INDEX	Region 1, Region 2, Region 3, Region 4, Φ mean, Ψ mean, Φ prob., Ψ prob.
THREE-DIMENSIONAL MOLECULAR DESCRIPTORS	PNSA ^1^, DPSA ^1^, PPSA ^1^, FNSA ^1^, FPSA ^1^, WNSA ^1^, WPSA ^1^, RNCS ^1^, RPCS ^1^, TASA ^1^, TPSA ^1^, RASA ^1^, RPSA ^1^, Diameter3D ^2^, Radius3D ^2^, GeometricalShapeIndex ^2^, PetitjeanIndex3D ^2^, MomentOfInertia ^2^, PBF ^2^
PLEC FINGERPRINTS	PLEC fingerprints, peptide length dummies
RESCORE+ SCORES	CHARMM ^3^, APBS ^4^, Elect ^4^, ElectDD ^4^, RPSCORE ^5^, MLPInS ^6^, MLPInS2 ^6^, MLPInS3 ^6^, MLPInSF ^6^, ChemPlp ^3^, XScore ^3^

^1^ Surface area descriptors; ^2^ shape-related descriptors; ^3^ intermolecular interaction energy scores; ^4^ electrostatic interaction energy scores; ^5^ protein–protein binding affinity scores; ^6^ hydrophobic interaction scores.

## Data Availability

Data for this article, including the curated dataset and the code to run PepScorer::RMSD, are available at Zenodo https://doi.org/10.5281/zenodo.17649889.

## Supplementary Materials

The following supporting information can be downloaded at https://www.mdpi.com/article/10.3390/ijms27020870/s1.

## Abbreviations

The following abbreviations are used in this manuscript:

AD, Applicability Domain; ADCP, Autodock Crank Pep; AF2, AlphaFold 2, BB-RMSD, BackBone atom Root-Mean-Squared Deviation; CV, Cross-Validation; DP, Docking Power; FFT, Fast-Fourier-Transform; FPs, Fingerprints; GB, Gradient Boosting; GNN, Graph Neural Network; HA-RMSD, Heavy-Atom Root-Mean-Squared Deviation; HGB, Histogram Gradient Boosting; I-RMSD, Interface Root-Mean-Squared Deviation; MAE, Mean Absolute Error; MD, Mahalanobis Distance; ML, Machine Learning; ODDT, Open Drug Discovery Toolkit; PDB, Protein Data Bank; PDF, Probability Density Function; PLEC, Protein–Ligand Extenced Connectivity; PPIs, Protein–Protein Interactions; RF, Random Forest; RMSD, Root-Mean-Squared Deviation; SFS, Sequential Feature Selection; SVM, Support Vector Machine; SVR, Support Vector Regression.
