# Supplementary material for: PepScorer::RMSD: An Improved Machine Learning Scoring Function for Protein–Peptide Docking
Source: Int J Mol Sci. 2026 Jan 15;27(2):870. doi: 10.3390/ijms27020870 (PMC12842220; doi:10.3390/ijms27020870)
Supplement: Supplementary file 1 [file ijms-27-00870-s001.zip › ijms-4069770-supplementary.pdf]

Figure S1. **Heatmap of the dataset similarly measured by TM-score.** The software MM-align performed a structural alignment of the protein binding pockets defined by a 10 Å sphere around the peptide ligand. The TM-score is a similarity metric normalized by the length of the longest chain and takes values in the range 0-1. The peptide-protein complexes are reported in the heatmap as grouped by a graph clustering procedure using a threshold of 0.4 TM-score, so that protein binding pockets sharing more than 0.4 of structural similarity are placed in the same group.

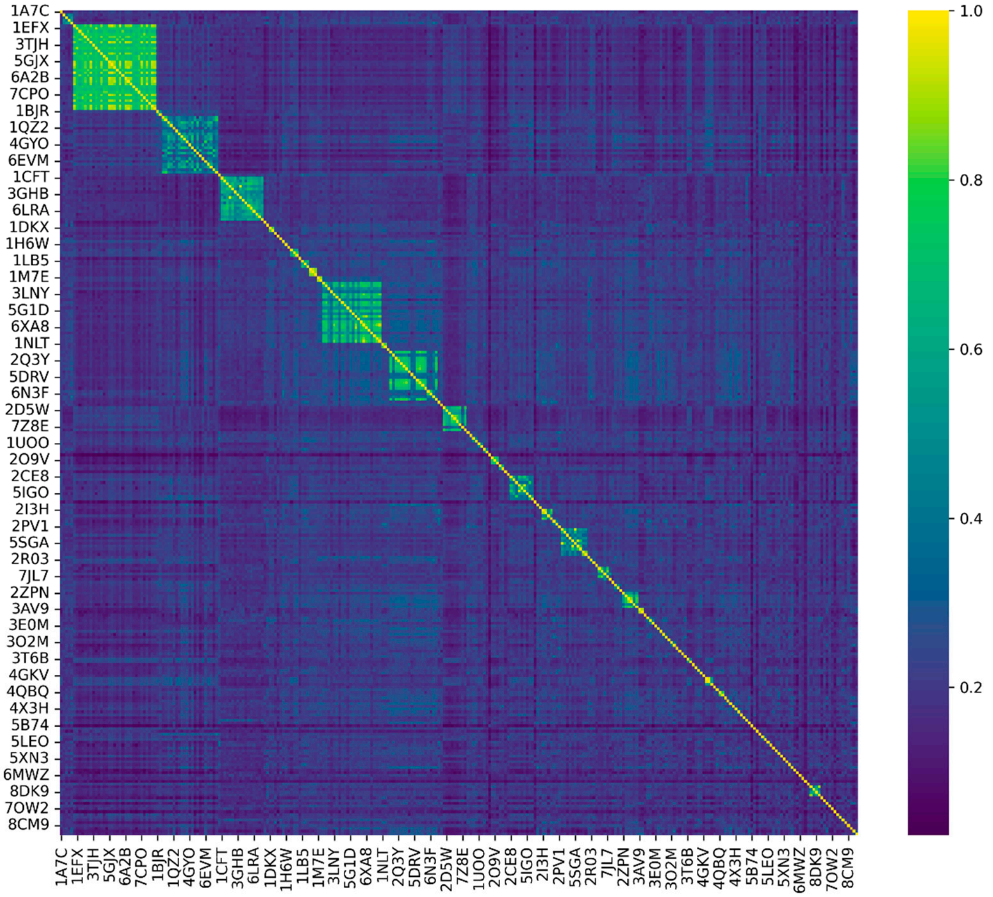

Figure S2. Correlation heatmap for the Ramachandran index features and RMSD values. The correlation is measured by the Pearson correlation coefficient R calculated between the two considered variables. As we can see, both Region 1 and psi prob. show an appreciable correlation with the RMSD of -0.44 and -0.48, respectively.

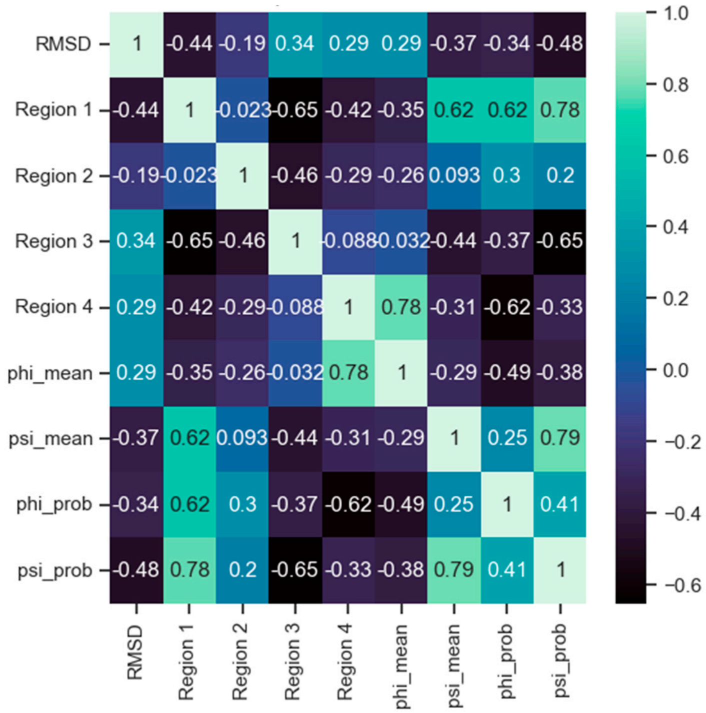

Section S1. Model performance as a function of peptide sequence identity.

To better explore the performance of our model as a function of peptide sequence identity, we decided to perform an experiment in which we removed from the evaluation set those complexes with a peptide ligand that shares a sequence identity higher than a certain threshold with any of the training peptides. To achieve this, we performed a pairwise sequence alignment of all the evaluation peptides against all the training peptides. The alignment was carried out with the function "globalds" of Biopython's module "pairwise2.align". We employed the BLOSUM62 matrix as a substitution matrix with a gap opening penalty of -11 and a gap extension penalty of -1. Thresholds between 0.4 and 1.0 were tested, which implied an increase in the size of the evaluation set, parallel to an increase in similarity between training and test data. As shown in Figure S5, model performances, measured in terms of mean absolute error (MAE) and Pearson correlation coefficient (R), are not influenced by the peptide sequence identity, thus confirming the ability of PepScore::RMSD to generalize on unseen data.

Figure S3. PepScore::RMSD performance on the evaluation set as a function of peptide sequence identity. The bar plot shows model performances in terms of MAE and R achieved on different test sets obtained by removing from the original one peptides that share a sequence similarity higher than a threshold with any peptide in the training set. Thresholds between 0.4 and 1.0 were tested, and the number above each pair of bars indicates the number of complexes in the evaluation set. Model performance remains stable during the experiment.

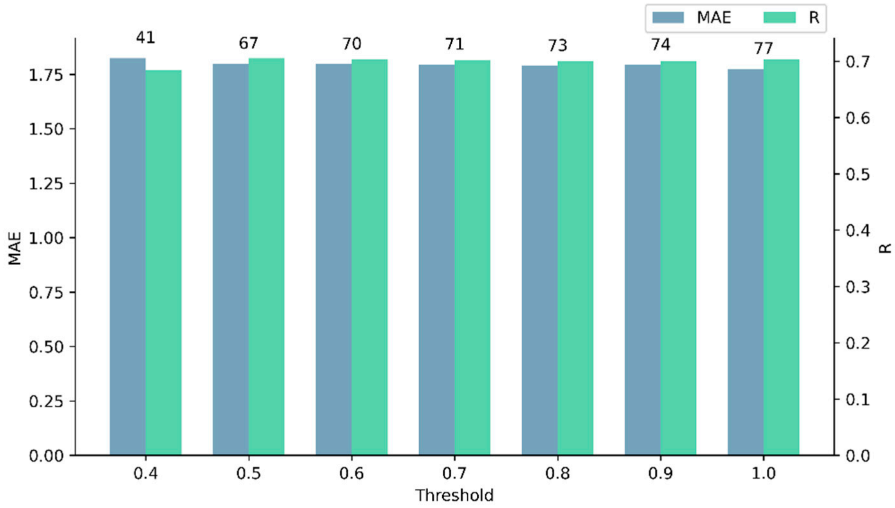

Figure S4. Docking power (DP) comparison between our workflow (PLANTS docking followed by PepScorer::RMSD poses re-ranking) and AlphaFold-Multimer. The full evaluation set includes the 77 complexes of subset used during internal evaluation and the restricted evaluation set includes 28 complexes containing peptides with six or fewer residues. When applied to the full evaluation set, AF2 outperforms the PLANTS plus PepScorer::RMSD workflow, achieving a top-1 DP of 28% while our method reaches up to 18% with ten poses (a). When applied to the restricted evaluation set, AF2's top-1 DP dropped to 21%, while our approach achieved a top-1 DP of 18% and reached 43% within the top-10 poses (b).

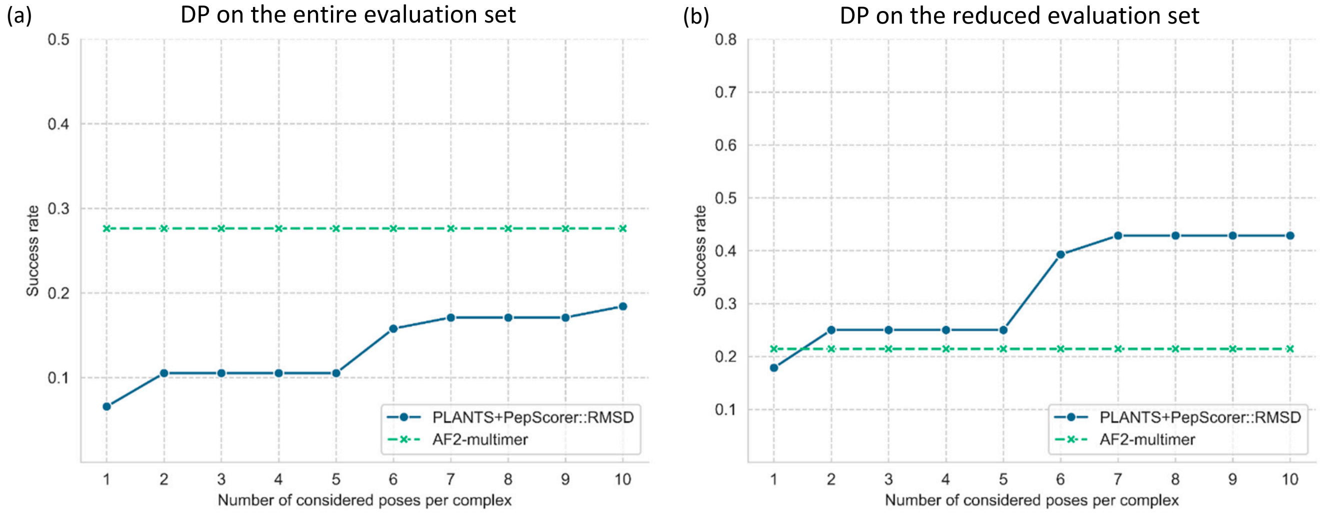

Figure S5. Docking power (DP) comparison on the low average pLDDT predictions between our workflow (PLANTS docking followed by PepScorer::RMSD poses re-ranking) and AlphaFold-Multimer. The low average pLDDT predictions is a reduced evaluation set (33 complexes) that includes only complexes for which the peptide's average pLDDT was lower than 70. Both methods achieved a top-1 DP of 9%, but PLANTS plus PepScorer::RMSD improved to a top-10 DP of 24%

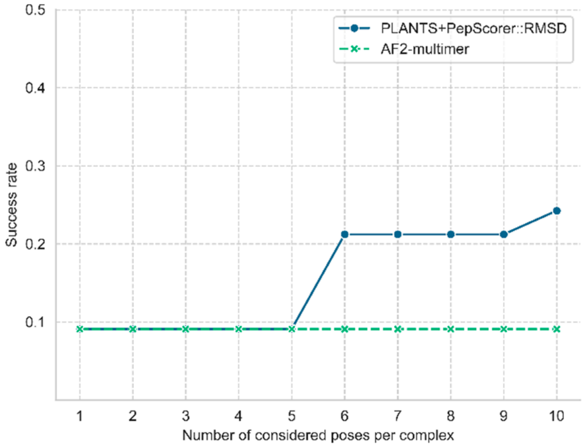

Figure S6. **RMSD values of the poses generated with energy minimization with respect to the x-ray binding conformation***.* *05lig*, in blue, refers to the pose obtained through an energy minimization of the complex, maintaining the protein fixed and the peptide with a harmonic restraint of 0.5, while *freelig* poses, in dashed green, were obtained leaving the peptide free to move. The complex 150 stands out as a clear outlier; therefore, it was removed from the dataset.

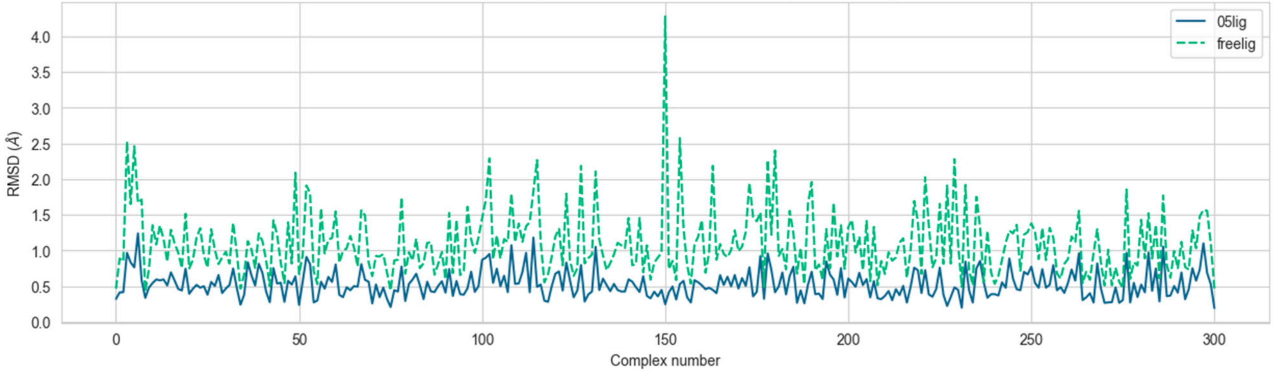

Figure S7. RMSD distribution in train and evaluation sets for peptide length. This boxplot reports the RMSD distribution in the train and evaluation splits, for each peptide length.

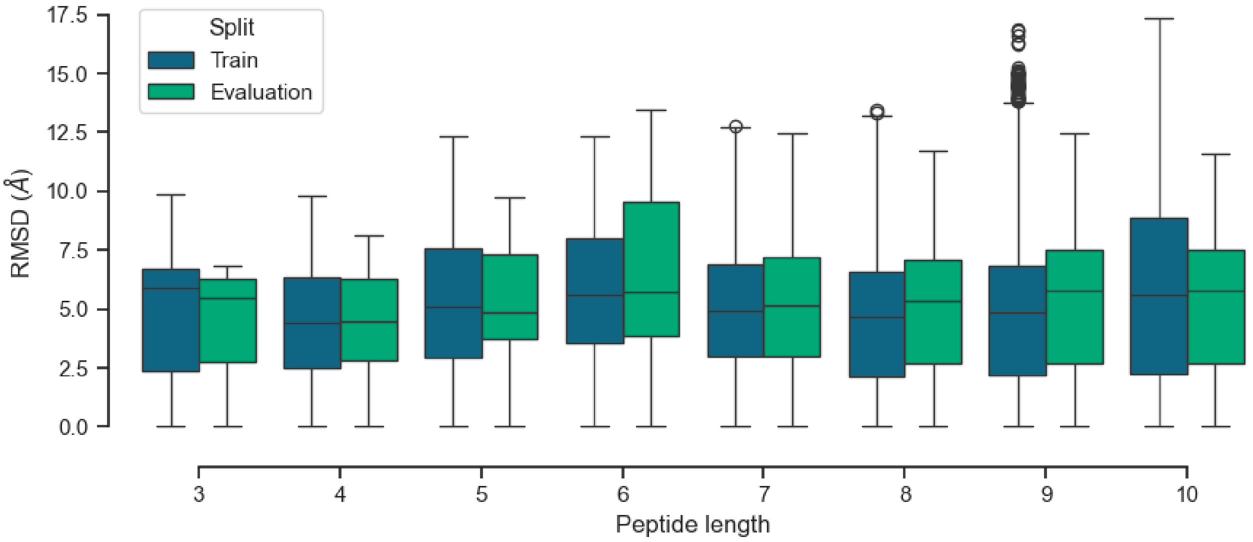

Table S1. Performance of models trained with full PLEC and PLEC selected with different variance thresholds (v.t.) 0.01, 0.1, 0.5. In each box, model performance is reported in terms of average Mean Absolute Error (MAE) (above) and Pearson correlation coefficient (R) (below), evaluated over 10-fold cross-validation. The box color scheme reflects the MAE values. Generally, low or no improvement in performance is observed by filtering the fingerprints with increasingly high thresholds. Support vector regressor (SVR) reported the best results for all the input matrices.

Feature set	Number of features	ElasticNet	Random Forest	Gradient boosting	Histogram gradient boosting	Support vector regression
PLEC	16384	2.60<br>0.18	2.52<br>0.29	2.53<br>0.28	2.55<br>0.35	2.41<br>0.41
PLEC v.t. 0.01	12294	2.60<br>0.18	2.53<br>0.28	2.53<br>0.30	2.55<br>0.34	2.41<br>0.41
PLEC v.t. 0.1	4335	2.60<br>0.18	2.51<br>0.31	2.48<br>0.34	2.52<br>0.36	2.41<br>0.40
PLEC v.t. 0.5	952	2.42<br>0.36	2.52<br>0.30	2.46<br>0.36	2.45<br>0.40	2.40<br>0.39

Table S2. Best model features sorted as obtained by the sequential forward feature selection (SFS). The correlation of the feature values and the RMSD values of all poses is reported in terms of Pearson coefficient R.

n.	Feature	Pearson R with the RMSD
1	psi_prob	-0.479482116
2	PLANTS_CHEMPLP_NORM_CONTACT	0.271527392
3	ElectDD	0.207347095
4	CHARMM	-0.009970121
5	APBS_Ligand	-0.126905881
6	RPScore_LigContacts	-0.115695795
7	MOMI-Z	0.253675017
8	DPSA1	0.053332183
9	PLANTS_CHEMPLP_NORM_WEIGHT	0.263029968
10	WNSA2:nrb	0.048037183
11	Region 1	-0.435204866
12	PLANTS_CHEMPLP	0.259871353
13	RPScore_Norm_LigContacts	-0.058943279
14	pep length_4	-0.095679705
15	XS_HPScore	-0.150986894
16	MOMI-Z:nrb	0.147252212
17	pep length_5	0.002468446
18	PNSA1:nrb	-0.173769944
19	pep length_3	-0.005639438
20	XS_HSScore	-0.182333359
21	RPCS:nrb	-0.156476116
22	PPSA1	0.053193659
23	RPScore_RecContacts	-0.118262691
24	PLANTS_CHEMPLP_RB_PEN_NORM_CRT_HEVATOMS	0.341553977
25	pep length_9	-0.008271558
26	PBF	0.180231473
27	pep length_8	-0.049552108
28	RPCS:nha	-0.102658456
29	pep length_10	0.085200306
30	RNCS:nrb	-0.102616537

Table S3. **The 147 selected 3D molecular descriptors calculated with Mordred**. Both raw and normalized descriptors are included in the table. The normalization was done by dividing the raw values by the number of rotatable bonds (nrb) and the number of heavy atoms (nha). The 46 descriptors selected after the correlation analysis carried out during model selection are highlighted in bold.

**PNSA1**	WNSA1	**PNSA1:nrb**	PPSA4:nha	**WNSA1:nrb**	TPSA:nha
PNSA2	WNSA2	PNSA2:nha	PPSA4:nrb	WNSA2:nha	TPSA:nrb
**PNSA3**	WNSA3	**PNSA2:nrb**	PPSA5:nha	**WNSA2:nrb**	RASA:nha
PNSA4	WNSA4	PNSA3:nha	PPSA5:nrb	WNSA3:nha	RASA:nrb
PNSA5	WNSA5	**PNSA3:nrb**	**FNSA1:nha**	WNSA3:nrb	RPSA:nha
**DPSA1**	WPSA1	PNSA4:nha	**FNSA1:nrb**	WNSA4:nha	RPSA:nrb
**DPSA2**	WPSA2	PNSA4:nrb	FNSA2:nha	WNSA4:nrb	**GeomDiameter:nha**
**DPSA3**	WPSA3	PNSA5:nha	FNSA2:nrb	WNSA5:nha	GeomDiameter:nrb
DPSA4	WPSA4	PNSA5:nrb	FNSA3:nha	WNSA5:nrb	GeomRadius:nha
DPSA5	WPSA5	DPSA1:nha	FNSA3:nrb	WPSA1:nha	GeomRadius:nrb
**PPSA1**	**RNCS**	**DPSA1:nrb**	FNSA4:nha	WPSA1:nrb	**GeomShapeIndex:nha**
PPSA2	**RPCS**	DPSA2:nha	FNSA4:nrb	WPSA2:nha	GeomShapeIndex:nrb
PPSA3	**TASA**	**DPSA2:nrb**	FNSA5:nha	WPSA2:nrb	GeomPetitjeanIndex:nha
PPSA4	TPSA	**DPSA3:nha**	FNSA5:nrb	WPSA3:nha	GeomPetitjeanIndex:nrb
PPSA5	**RASA**	**DPSA3:nrb**	**FPSA1:nha**	WPSA3:nrb	MOMI-X:nha
**FNSA1**	RPSA	**DPSA4:nha**	**FPSA1:nrb**	WPSA4:nha	MOMI-X:nrb
FNSA2	**GeomDiameter**	**DPSA4:nrb**	FPSA2:nha	WPSA4:nrb	MOMI-Y:nha
FNSA3	GeomRadius	DPSA5:nha	FPSA2:nrb	WPSA5:nha	MOMI-Y:nrb
FNSA4	**GeomShapeIndex**	DPSA5:nrb	**FPSA3:nha**	WPSA5:nrb	MOMI-Z:nha
FNSA5	GeomPetitjeanIndex	**PPSA1:nha**	**FPSA3:nrb**	**RNCS:nha**	**MOMI-Z:nrb**
FPSA1	MOMI-X	PPSA1:nrb	FPSA4:nha	**RNCS:nrb**	**PBF:nha**
FPSA2	MOMI-Y	PPSA2:nha	FPSA4:nrb	**RPCS:nha**	**PBF:nrb**
**FPSA3**	**MOMI-Z**	PPSA2:nrb	FPSA5:nha	**RPCS:nrb**	
FPSA4	**PBF**	PPSA3:nha	FPSA5:nrb	**TASA:nha**	
FPSA5	**PNSA1:nha**	**PPSA3:nrb**	WNSA1:nha	TASA:nrb	

Table S4. The 30 selected Rescore+ scores.

APBS_Ligand	PLANTS_CHEMPLP_NORM_WEIGHT
APBS_Receptor	PLANTS_CHEMPLP_NORM_CRT_WEIGHT
APBS_Complex	PLANTS_CHEMPLP_RB_PEN_NORM_CRT_HEVATOMS
APBS_Binding	PLANTS_CHEMPLP_NORM_CONTACT
CHARMM	RPScore
Elect	RPScore_Norm_LigAA
ElectDD	RPScore_Norm_LigContacts
MlpInS	RPScore_LigContacts
MlpInS2	RPScore_RecContacts
MlpInS3	RPScore_TotContacts
MlpInSF	XS_HPScore
PLANTS_CHEMPLP	XS_HMScore
PLANTS_CHEMPLP_RB_PEN	XS_HSScore
PLANTS_CHEMPLP_NORM_HEVATMS	XS_Average
PLANTS_CHEMPLP_NORM_CRT_HEVATMS	XS_Binding

Table S5. Optimized hyperparameters and relative search spaces. The hyperparameter tuning was conducted with the GridSearch method from scikit-learn. Therefore, every possible combination of the parameters proposed is evaluated. The tuning was done for the top-3 algorithms in terms of average Mean Absolute Error (MAE) in 10-fold cross-validation.

Algorithm	Parameter	Values
Random Forest	n_estimators	50; 100; 1000
	max_features	0.2; 0.4; 0.6; 0.8; None
	max_leaf_nodes	2; 10; 40; 70; 100; None
Gradient Boosting	n_estimators	10; 50; 100; 1000
	learning_rate	0.01; 0.05; 0.1; 0.5; 1
	max_leaf_nodes	2; 10; 40; 70; 100
Histogram Gradient Boosting	max_iter	10; 50; 100; 1000
	learning_rate	0.01; 0.05; 0.1; 0.5; 1
	max_leaf_nodes	2; 10; 40; 70; 100
